# Outcome of Percutaneous Coronary Intervention in Old Patients Presenting with Acute Coronary Syndrome

**DOI:** 10.34172/aim.2022.84

**Published:** 2022-08-01

**Authors:** Aida Fallahzadeh, Ali Sheikhy, Afsaneh Aein, Mojtaba Salarifar, Hamidreza Pourhosseini, Hassan Aghajani, Mohammad Alidoosti, Saeed Sadeghian, Kaveh Hosseini

**Affiliations:** ^1^Tehran Heart Center, Tehran University of Medical Sciences, Tehran, Iran; ^2^Non-Communicable Disease Research Center, Endocrinology and Metabolism Population Sciences Institute, Tehran University of Medical Sciences, Tehran, Iran; ^3^Department of Health Education and Health Promotion, School of Public Health, Iran University of Medical Sciences, Tehran, Iran; ^4^Department of Cardiology, Tehran Heart Center, Tehran University of Medical Sciences, Tehran, Iran

**Keywords:** Elderly, Major adverse cardiac events, Octogenarian, Percutaneous coronary intervention

## Abstract

**Background:**

Octogenarians (age≥80 years) with coronary artery disease constitute a high-risk group and the elderly undergoing percutaneous coronary intervention (PCI) are at higher risk of adverse outcomes compared to young patients. In this study, we aimed to describe the outcomes of the elderly with acute coronary syndrome (ACS) who underwent PCI and also to identify the predictors of short-term major adverse cerebrocardiovascular events (MACCE) in octogenarians.

**Methods:**

In this registry-based cohort study, we reviewed the data of patients (aged≥65 years) who underwent PCI. Univariate Cox-regression model was used to assess the univariate effects of covariates on mortality and MACCE and multivariate Cox-regression analysis were used to discover MACCE predictors.

**Results:**

We reviewed the data of 3332 patients (2722 elderly [65 to 79 years], and 610 octogenarians [≥80 years]). The cumulative hazard of MACCE was significantly higher in the octogenarian group compared with the younger group (*P*<0.001). MACCE in octogenarians presenting with ST-elevation myocardial infarction (STEMI) was significantly higher than those with non-ST-elevation myocardial infarction/Unstable angina (NSTEMI/UA) (*P*<0.001); however, the cumulative hazard of mortality was not significantly different between the two groups (*P*=0.270). Successful PCI, left main stenosis and estimated glomerular filtration rate (eGFR) were independent predictors of MACCE in octogenarians with ACS.

**Conclusion:**

Octogenarians undergoing PCI had a higher rate of MACCE and mortality compared with a younger population. In octogenarians, MACCE in those with STEMI was significantly higher than those with NSTEMI/UA and the mortality trend was similar; however, the 1-year trend was in favor of the STEMI subgroup.

## Introduction

 With the increasing life expectancy and falling mortality rate, the octogenarian population in Iran has been increasing sharply during the past decades. By the year 2050, it is expected to have almost 3.5 million persons aged 80 years and above, which account for 3.8% of the total population.^1^

 The prevalence of coronary artery disease is increasing in the elderly and cardiovascular disease has been established in about 80% of octogenarian population.^2,3^ With advancing population age, elderly patients with acute coronary syndrome (ACS) (ST segment elevated myocardial infarction [STEMI], non-ST segment elevated myocardial infarction [NSTEMI] and unstable angina [UA]) will represent a great proportion of patients undergoing percutaneous coronary intervention (PCI).^4^ Although PCI is associated with improved survival in octogenarians compared with medical therapy alone, the elderly undergoing PCI are at higher risk of adverse outcomes and mortality compared to young patients.^5^ This emphasizes the importance of identifying prognostic factors associated with adverse outcomes after PCI in octogenarians.

 Although studies have shown that mortality is higher in older patients undergoing PCI compared to young individuals,^6,7^ the differences in PCI outcomes between the elderly (65–79 years) and octogenarians ( ≥ 80 years) have not been well described. Moreover, comparing outcomes among octogenarians presenting with STEMI or NSTEMI/UA is another issue that has not been clearly addressed in previous studies. Finding the perioperative risk factors which affect the outcome is necessary since they could influence clinical decision and patient selection.^8^ Several well-designed clinical trials have been conducted previously to establish the outcome of PCI in elderly patients,^9,10^ but clinical trials are not capable to determine predictors.

 In this registry-based cohort study, we aimed to evaluate the short-term outcome of PCI in patients aged 65–79 years and ≥ 80 years. Moreover, we identified the independent predictors of major adverse cerebrocardiovascular events (MACCEs) in octogenarians presenting with STEMI or NSTEMI/UA.

## Materials and Methods

###  Study Design

 In accordance with the statement checklist outlined in Strengthening the Reporting of Observational Studies in Epidemiology (STROBE), we conducted a registry-based, single-center cohort study in Tehran Heart Center (THC).^11^ Patient characteristics and procedural details were recorded prospectively at the time of procedure and afterwards during each follow-up visit. The THC ethical board approved the study (IR-THC-13 799). This study did not meet the criteria for informed consent; consequently, an “informed consent waiver” was obtained from the THC ethical board.

###  Study Population

 The entire cohort included 8126 patients with ACS who underwent coronary angiogram and PCI between April 2015 and December 2019. Our inclusion criteria were: 1) age ≥ 65 years; 2) adequate pre-operative characteristics and 3) completion of follow-up visits. The main exclusion criterion was incomplete clinical and angiographic data. Eventually, 3332 patients remained in the study. The clinical follow-up data were collected by regular clinic visits (64.3% of patients) or direct telephone interviews (33.0% of patients). The patients’ follow-up protocol was at 4th, 6th, and 12th month after PCI and annually thereafter. Patients were stratified into two groups; 65 to 79 years old (elderly), which included 2722 (81.69%) patients, and ≥ 80 years old (octogenarian), which included 610 (18.31%) patients (Figure 1). The octogenarian group included all patients aged ≥ 80 years^12^ at the time of PCI, and patients did not switch groups if they passed the age of 80 years during the follow-up period.

**Figure 1:**
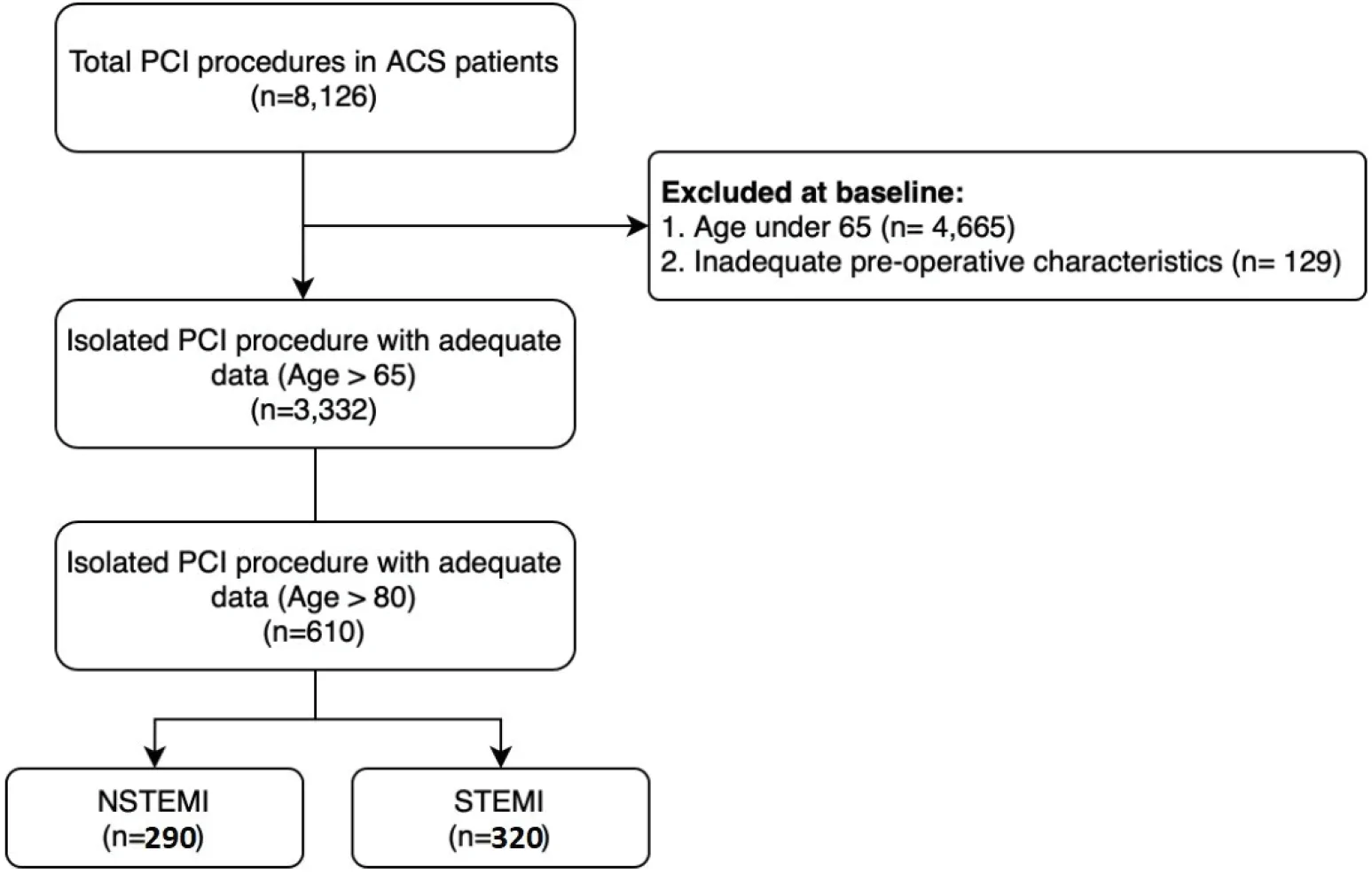
Flow Diagram of the Study Population

###  Definition of Variables 

 Data of the study population were retrieved from the angiography/PCI databank of our center.

 ACS refers to a spectrum of clinical presentations ranging from those for STEMI to presentations found in NSTEMI or in UA. The definition of hypertension was based on the presence of at least one of the following conditions; minimum systolic blood pressure of 140 mm Hg, or minimum diastolic blood pressure of 90 mm Hg, or history of taking antihypertensive drugs.^13^ The definition of dyslipidemia was based on the presence of a minimum total cholesterol of 240 mg/dL, a minimum triglyceride of 200 mg/dL, a high-density lipoprotein cholesterol < 40 mg/dL in men and < 50 mg/dL in women, a minimum low-density lipoprotein cholesterol level of 160 mg/dL, or history of taking lipid-lowering drugs.^14,15^ We determined cigarette smoking and opium usage based on patient’s self-reported status. A current smoker was a person who currently smokes and has ever smoked more than 100 cigarettes.^16^ Based on the American Diabetes Association, diabetes mellitus was defined as the presence of a definite history of diabetes with records of treatment or fasting blood sugar ≥ 126 mg/dL or 2-hour post-prandial glucose ≥ 200 mg/dL.^17^ Positive family history was defined as a first-degree relative younger than 55 years (men) or 65 years (women) with coronary artery disease. Infarct-related artery was the artery that underwent stent implantation during the PCI procedure. The thrombolysis in myocardial infarction (TIMI) flow grade is a method for the assessment of coronary artery flow in ACS. It is classified as grade 0 (no flow), grade 1 (penetration without perfusion), grade 2 (partial perfusion) or grade 3 (complete perfusion). Left main (LM) stenosis referred to those with stenosis of the left main coronary artery < 50% on angiogram.

###  Procedural Technique

 The standard techniques with the femoral approach were applied for performing PCI procedures. According to our procedure routine, before the procedure, all patients received a 300–600 mg loading dose of clopidogrel plus 325 mg aspirin. During the procedure, patients received 70–100 IU/kg intravenous unfractionated heparin. Clopidogrel (75 mg/d) and aspirin (81 mg/d) were maintained for at least 1-year after the procedure.

###  Study Endpoints

 All-cause mortality and MACCE occurrence (including all-cause mortality, non-fatal ACS, non-fatal stroke or transient ischemic attack, and repeat coronary revascularization via PCI or Coronary artery bypass graft surgery) were considered as primary endpoints.

####  Statistical Analysis 

 Normally and skewed distributed continuous variables were presented as mean with standard deviation (SD) and median with 25th and 75th percentiles (interquartile range [IQR] boundaries), respectively. Histogram charts as well as central tendency and dispersion measures were used to assess the normality of variables. Student’s *t* test and Mann-Whitney U-test were used to compare normally and skewed distributed variables, respectively. Discrete variables were presented as frequency and percentages, and compared between groups using the Chi-square test.

 A univariate Cox regression model was used to assess the univariate effects of covariates on mortality and MACCE, and reported as hazards ratio (HR) with 95% confidence intervals (CIs). We entered covariates with *P* values < 0.1 on the univariate Cox regression into the multivariate model. The decision of what to include in the multivariable models was based on an *a priori* hypothesis and if adding/removing a variable made a big change (e.g., > 10%) in the estimates. Moreover, for each final variable, we tested the proportional hazard (PH) assumption through graphical assessment based on the scaled Schoenfeld residuals. The assumption was met for each variable conclusively (Supplementary file 1, Figure S1).

 We used the Stata Statistical Software, release 14 (College Station, TX: StataCorp LP) and R version 4.0.3 for all statistical analyses.^18^ The following packages in R were used: “survival” (package for survival analysis in R),^19^ “survminer” (drawing survival curves),^20^ and “ggplot2”.^21^

## Results

###  Patient Characteristics 

 The baseline and procedural characteristics of the study population are shown in Table 1. In the STEMI subgroup, the proportion of female patients was 36.7% in octogenarians, significantly higher than the elderly (*P* = 0.001), and the median age was 83 [81-89] and 70 [67-75] years, respectively. Procedural characteristics were not different between the two groups except for stent length and diameter.

**Table 1:** Baseline and Procedural Characteristics by Age

	**STEMI**			**NSTEMI**		
	**65–80 Years**	**≥80 Years**	***P*** ** Value**	**65–80 Years**	**≥80 Years**	***P*** ** Value**
Total number	1163 (78.59%)	320 (21.41%)	< 0.001	1559 (84.13%)	290 (15.87%)	< 0.001
Female	348 (30.0%)	116 (36.7%)	0.001	549 (35.2%)	93 (31.6%)	0.088
Age (median, IQR) (y)	70 [67–75]	83 [81–89]	< 0.001	70 [67–75]	83 [81–86]	< 0.001
BMI (mean, SD) (kg/m^2^)	27.58 (4.41)	26.35 (4.76)	< 0.001	27.96 (4.66)	26.64 (4.86)	< 0.001
eGFR (median, IQR) (mL/min)	65.91 [51.13–83.48]	42.31 [32.11–57.84]	< 0.001	64.64 [51.85–79.82]	45.26 [34.41–58.27]	< 0.001
LVEF (mean, SD) (%)	41.17 (8.78)	38.77(9.08)	< 0.001	46.83 (8.90)	43.85 (10.34)	< 0.001
Family history	128 (11.0%)	22 (7.0%)	0.001	204 (13.1%)	30 (10.2%)	0.043
Dyslipidemia	608 (52.4%)	127 (40.2%)	< 0.001	950 (60.9%)	140 (47.6%)	< 0.001
Previous MI	67 (5.6%)	21 (6.6%)	0.356	578 (38.1%)	137 (47.2%)	< 0.001
Hypertension	678 (58.4%)	198 (62.7%)	0.049	971 (62.3%)	195 (66.3%)	0.062
Diabetes mellitus	559 (48.2%)	132 (41.8%)	0.004	616 (39.5%)	98 (33.3%)	0.004
Smoking	414 (35.7%)	72 (22.8%)	< 0.001	510 (32.7%)	63 (21.4%)	< 0.001
Opium consumption	153 (13.2%)	20 (6.3%)	< 0.001	171 (11.0%)	21 (7.1%)	0.002
Cerebrovascular disease	68 (5.9%)	19 (6.0%)	0.925	53 (3.4%)	19 (6.5%)	0.001
COPD	31 (2.7%)	12 (3.8%)	0.165	54 (3.5%)	8 (2.7%)	0.302
Infarct-related artery						
Left main artery	6 (0.5%)	0	0.254	17 (1.1%)	6 (2.1%)	0.011
Left anterior descending artery	556 (46.2%)	170 (53.1%)		670 (44.1%)	154 (53.1%)	
Left circumflex artery	218 (18.1%)	49 (15.3%)		322 (21.2%)	55 (19%)	
Right coronary artery	361 (30.0%)	83 (25.9%)		366 (24.1%)	48 (16.6%)	
Saphenous vein graft	52 (4.3%)	13 (4.1%)		129 (8.5%)	25 (8.6%)	
Multivessel disease	823 (70.9%)	227 (71.8%)	0.656	1031 (66.1%)	208 (70.7%)	0.027
Left main stenosis	105 (9%)	40 (12.6%)		118 (7.5%)	30 (10.2%)	
Preprocedural TIMI flow						
0	706 (60.9%)	187 (59.2%)	0.333	161 (10.3%)	24 (8.2%)	0.422
1	66 (5.7%)	16 (5.1%)		40 (2.6%)	6 (2.0%)	
2	200 (17.2%)	68 (21.5%)		168 (10.8%)	39 (13.3%)	
3	188 (16.2%)	45(14.2%)		1190 (76.3%)	225 (76.5%)	
Postprocedural TIMI flow						
0	41 (3.5%)	14 (4.4%)	0.241	34 (2.2%)	8 (2.7%)	0.599
1	7 (0.6%)	5 (1.6%)		6 (0.4%)	1 (0.3%)	
2	66 (5.7%)	14 (4.4%)		22 (1.4%)	7 (2.4%)	
3	1046 (90.2%)	283 (89.6%)		1497 (96.0%)	278 (94.6%)	
ACC/AHA type						
A	2 (0.2%)	0	0.253	2 (0.1%)	0	0.558
B1	82 (7.1%)	23 (7.3%)		383 (24.6%)	77 (26.1%)	
B2	166 (14.3%)	55 (17.4%)		219 (14.0%)	40 (13.6%)	
C	910 (78.4%)	238 (75.3%)		955 (61.3%)	177 (60.2%)	
Stent diameter (median, IQR) (mm)	2.75 [2.5-3]	3 [2.75, 3]	< 0.001	3 [2.75, 3.25]	3 [2.75, 3.5]	0.409
Stent length (median, IQR) (mm)	24 [19, 33]	28 [20, 38]	0.024	24 [18, 33]	23 [18, 32]	0.731
Stent thrombosis	21 (1.8%)	5 (1.6%)	0.119	5 (0.3%)	0	< 0.001

BMI, Body mass index; eGFR, estimated glomerular filtration rate; LVEF, left ventricular ejection fraction; MI, myocardial infarction; COPD, chronic obstructive pulmonary disease; TIMI, thrombolysis in myocardial infarction.

 In the NSTEMI subgroup, the median age was 83 [81-86] years in octogenarian and 70 [67-75] years in elderly patients. Multivessel disease was significantly higher in the octogenarian group compared with younger individuals (70.7% vs. 66.1%, *P*= 0.027).

###  Outcomes

 The occurrence of study endpoints in these two groups is shown in Table 2.

**Table 2:** Patients’ Outcomes by Age

	**Total**	**65-79 years**	**≥80 years**	***P *** **Value**
MACCE	509 (15.28%)	385 (14.14%)	124 (20.33%)	< 0.001
Death	147 (4.41%)	66 (2.42%)	81 (13.28%)	< 0.001
ACS/Revascularization	361 (10.83%)	318 (11.68%)	43 (7.49%)	0.003
Stroke/TIA	1 (0.03%)	1 (0.04%)	0	0.465

 According to Kaplan–Meier analysis, the cumulative hazard of MACCE was significantly higher in the octogenarian group in comparison with younger patients (median follow-up 12.06 months [IQR: 7.36-13.23 months], *P* < 0.001) (Figure 2A). The Kaplan-Meier survival curve demonstrated that survival was significantly lower in octogenarians compared to the elderly (*P* < 0.001) (Figure 2B).

**Figure 2:**
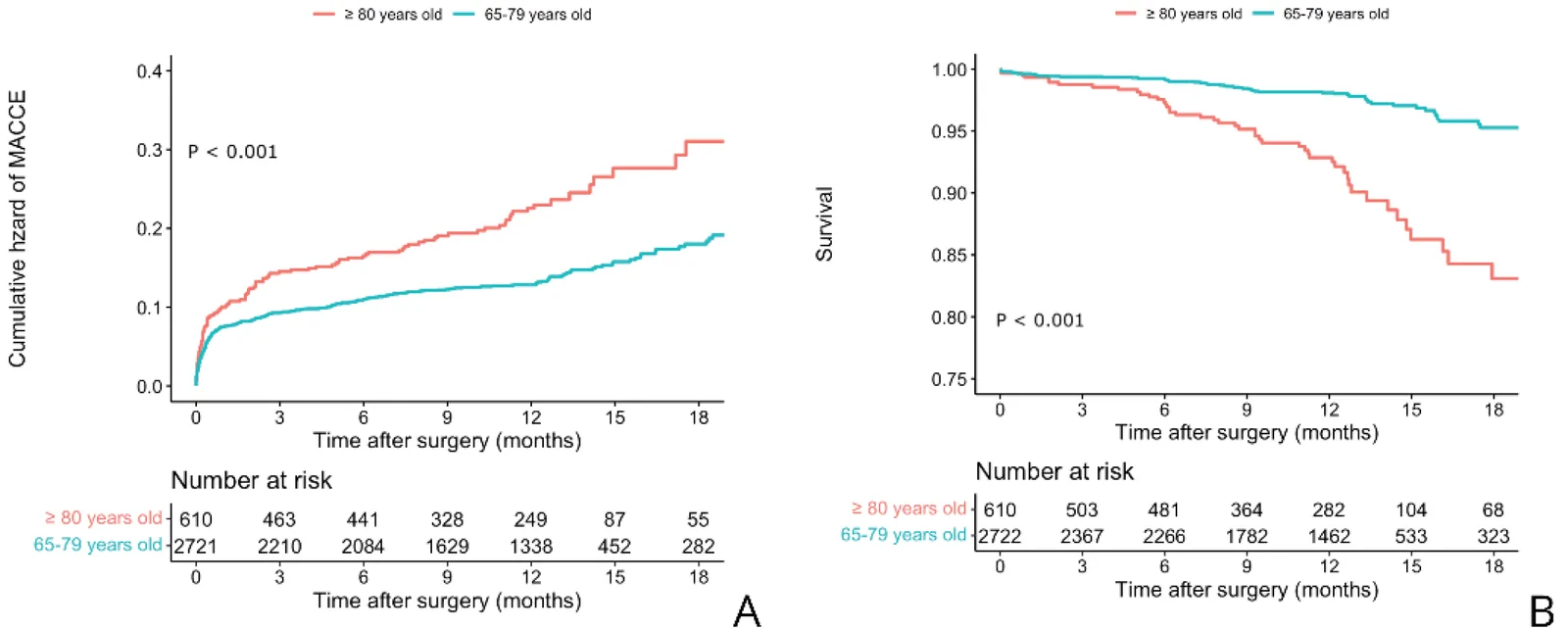
Kaplan–Meier Curves Showing Cumulative Hazard of (A) MACCE and (B) Survival in Octogenarians and the Elderly

###  Octogenarian Subgroup Analysis

 The baseline and procedural characteristics of the octogenarian subgroup based on the occurrence of MACCE are shown in Table 3.

**Table 3:** Patients’ Characteristics in the Octogenarian Group ( ≥ 80 years) by MACCE Occurrence

	**STEMI**			**NSTEMI**		
	**MACCE+**	**MACCE -**	***P *** **Value**	**MACCE+**	**MACCE -**	***P *** **Value**
Total number	77	243		47	243	
Female	28 (36.4%)	90 (37.0%)	0.915	13 (27.7%)	78 (32.1%)	0.548
Age (mean, SD) (y)	85.13 (4.4)	84.26 (3.8)	0.099	83.66 (3.6)	83.57 (3.1)	0.860
BMI (mean, SD) (kg/m^2^)	26.88 (5.2)	26.17 (4.6)	0.279	26.37 (4.3)	26.67 (4.9)	0.700
eGFR (mean, SD) (mL/min)	46.31 (18.0)	51.48 (17.6)	0.034	45.87 (18.1)	54.18 (19.3)	0.007
LVEF (mean, SD) (%)	36.43 (10.5)	38.15 (11.05)	0.262	34.45 (16.4)	41.81 (14.1)	0.002
Family history	6 (7.8%)	17 (7.0%)	0.814	5 (10.6%)	24 (9.9%)	0.795
Dyslipidemia	30 (39.0%)	98 (40.3%)	0.831	15 (31.9%)	124 (51%)	0.016
Previous MI	3 (3.9%)	18 (7.4%)	0.428	21 (44.7%)	116 (47.4%)	0.701
Hypertension	48 (62.3%)	153 (63%)	0.921	32 (68.1%)	160 (65.8%)	0.766
Diabetes mellitus	40 (51.9%)	94 (38.7%)	0.043	18 (38.3%)	78 (32.1%)	0.408
Smoking	15 (19.5%)	57 (23.5%)	0.467	11 (23.4%)	52 (21.4%)	0.760
Opium consumption	4 (5.2%)	16 (6.6%)	0.792	1 (2.1%)	20 (8.2%)	0.217
Cerebrovascular disease	5 (6.5%)	14 (5.8%)	0.781	2 (4.3%)	17 (7%)	0.748
COPD	1 (1.3%)	11 (4.5%)	0.306	2 (4.3%)	6 (2.5%)	0.620
Infarct-related artery						
Left main artery	0	0	0.087	3 (6.4%)	3 (1.2%)	0.047
Left anterior descending artery	44 (57.1%)	126 (51.9%)		19 (40.4%)	135 (55.6%)	
Left circumflex artery	17 (22.1%)	32 (13.2%)		10 (21.3%)	45 (18.5%)	
Right coronary artery	13 (16.9%)	70 (28.8%)		7 (14.9%)	41 (16.9%)	
Saphenous vein graft	3 (3.9%)	10 (4.1%)		7 (14.9%)	18 (7.4%)	
Multivessel disease	56 (72.7%)	174 (71.6%)	0.849	39 (83%)	166 (68.3%)	0.043
Left main stenosis	14 (18.2%)	26 (10.7%)	0..084	11 (23.4%)	19 (7.8%)	0.001
Preprocedural TIMI flow						
0	50 (64.9%)	137 (56.4%)	0.356	8 (17%)	16 (6.6%)	0.054
1	5 (6.5%)	11 (4.5%)		0	6 (2.5%)	
2	14 (18.2%)	54 (22.2%)		8 (20.5%)	31 (12.8%)	
3	8 (10.4%)	41 (16.9%)		31 (66%)	190 (78.2%)	
Postprocedural TIMI flow						
0	7 (9.1%)	8 (3.3%)	< 0.001	5 (10.6%)	2 (0.8%)	< 0.001
1	2 (2.6%)	3 (1.2%)		1 (2.1%)	0	
2	9 (11.7%)	5 (2.1%)		3 (6.4%)	4 (1.6%)	
3	59 (76.6%)	227 (93.4%)		38 (80.9%)	237 (97.5%)	
ACC/AHA type						
A	0	1 (0.4%)	0.891	0	0	0.674
B1	5 (6.5%)	18 (7.4%)		11 (23.4%)	65 (26.7%)	
B2	15 (19.5%)	41 (16.9%)		5 (10.6%)	34 (14.0%)	
C	57 (74%)	183 (75.3%)		31 (66%)	144 (59.3%)	
Stent diameter (mean, SD) (mm)	2.77 (0.34)	2.87 (0.43)	0.106	3.05 (0.4)	2.96 (0.4)	0.302
Stent length (mean, SD) (mm)	27.07 (10.4)	27.05 (9.4)	0.990	25.13 (8.07)	25.06 (9.3)	0.971
Stent thrombosis	2 (2.6%)	3 (1.2%)	0.597	0	0	

BMI, Body mass index; eGFR, estimated glomerular filtration rate; LVEF, left ventricular ejection fraction; MI, myocardial infarction; COPD, chronic obstructive pulmonary disease; TIMI, thrombolysis in myocardial infarction.

 Kaplan–Meier analysis showed that the cumulative hazard of MACCE in octogenarians presenting with STEMI was significantly higher than those with NSTEMI/UA (*P* < 0.001); however, the cumulative hazard of mortality was not significantly different between the two groups (*P*= 0.270). Although the mortality trend looked similar in the first 6 months, the 1-year trend was in favor of the STEMI subgroup (Figures 3A and 3B).

**Figure 3:**
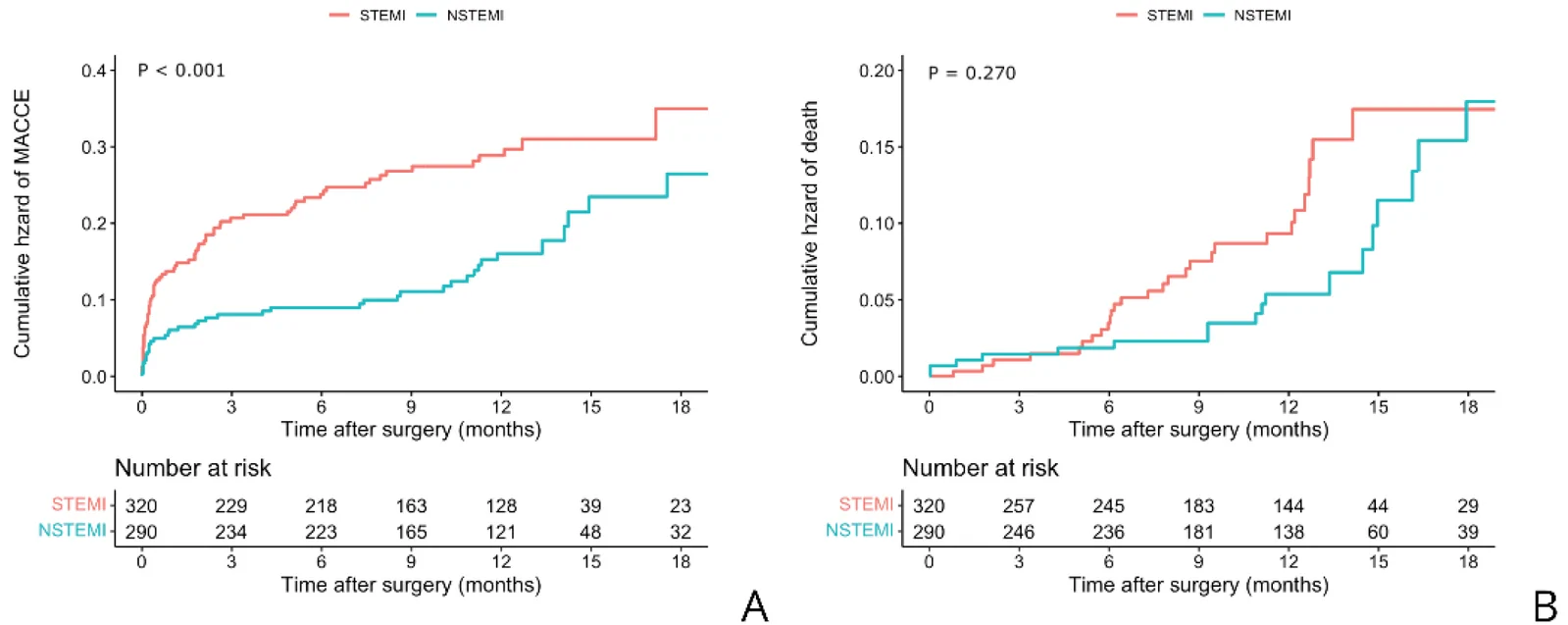
Kaplan–Meier Curves Showing Cumulative Hazard of (A) MACCE and (B) Death in Patients > 80 Years in the Two Groups (STEMI and NSTEMI/UA)

 The independent predictors of MACCE in octogenarians presenting with STEMI or NSTEMI/UA are shown in Figure 4 and Tables S1 and S2. In octogenarians presenting with STEMI; postprocedural TIMI flow 3 (HR 0.29 [95% CI: 0.16–0.53]), LM stenosis (HR 1.84 [95% CI: 0.92–3.66]) and estimated glomerular filtration rate (eGFR) (HR 0.98 [95% CI:0.96-0.99]) were the predictors, and in patients presenting with NSTEMI/UA; postprocedural TIMI flow 3 (HR 0.22 [95% CI: 0.10–0.49]), LM stenosis (HR 2.50 [95% CI: 1.17–5.34]), eGFR (HR 0.97 [95% CI: 0.95–0.99]), and dyslipidemia (HR 0.37 [95% CI: 0.19–0.72]) were the independent predictors of MACCE.

**Figure 4:**
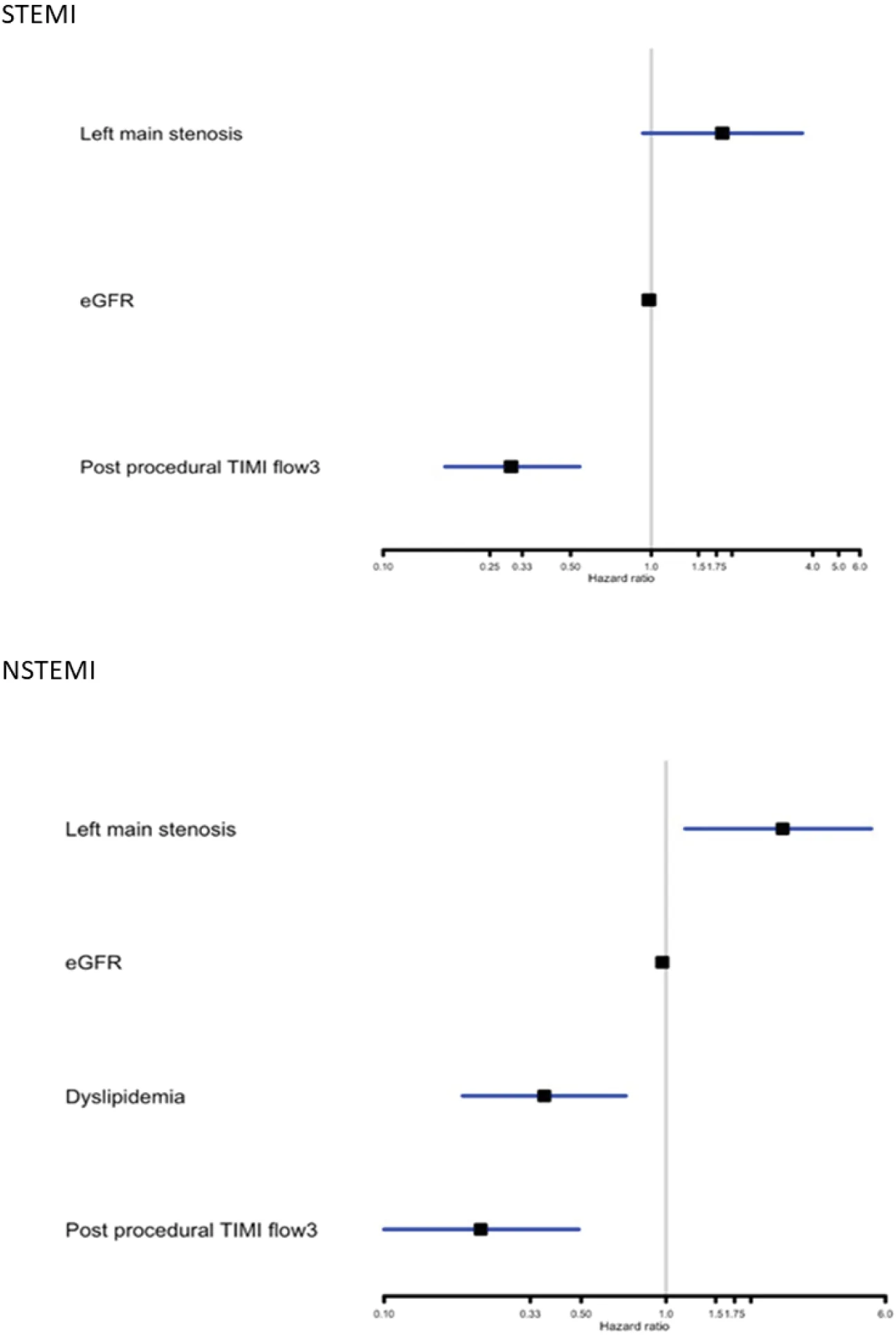
Final Predictors of MACCE in Patients > 80 Years in The Two Groups (STEMI and NSTEMI/UA)

## Discussion

 In the present study, we compared MACCE and mortality between the elderly and octogenarian subgroups and also identified the prognostic predictors of MACCE in the octogenarian population, one year after undergoing PCI due to ACS. We showed that MACCE and mortality were significantly higher in octogenarians compared with younger adults. Successfully performed PCI (defined as post-procedural TIMI flow 3) and higher level of eGFR were associated with lower risk of MACCE, and LM stenosis was associated with higher risk of MACCE in both STEMI and NSTEMI/UA groups.

 The pathophysiological mechanisms and clinical symptoms of ACS in octogenarians differ from those in younger patients. Octogenarians are known to have more complex lesions as well as multivessel disease.^6^ PCI is an accepted treatment choice for revascularization in the elderly.^22^ However, the higher presence of cardiac and non-cardiac diseases, worse endothelial function and increased calcification will lead to higher rates of adverse events after PCI in the elderly compared to young patients.^23^ As the elderly population is increasing globally due to advanced health care, evaluating the survival and identifying the predictors of MACCE in octogenarians after PCI becomes a more important issue.^24^ The higher risk of death in octogenarians is partly due to the presence of comorbid conditions such as hypertension, diabetes, ventricular dysfunction, heart failure, peripheral vascular disease and renal failure as well as procedural complications such as bleeding events, shock, and hemodynamic instability.^7,25,26^ Another explanation for the poor outcome in octogenarians is delayed reperfusion in the elderly due to late presentation and atypical symptoms.^27^ Yan et al evaluated 4,360 patients who underwent PCI and showed that octogenarians had significantly increased risk of mortality and major adverse cardiac events (MACE) at 30-days and 1-year after PCI compared to those aged < 80 years.^6^ Batchelor et al evaluated in-hospital outcomes and showed that octogenarians had increased risk of mortality and the composite outcome of death/myocardial infarction (MI)/cerebrovascular accident compared to those < 80 years.^7^ Fach et al assessed patients presenting with STEMI and showed that mortality and MACE rates significantly increased with age, as we reported in the present study.^28^ Feldman et al evaluated the effect of age on short-term prognosis after PCI in three age groups ( < 60, 60–80, and > 80 years) and showed that in both emergency and elective PCI groups, MACE increased with age.^29^

 In the present study, MACCE was significantly higher in octogenarians with STEMI compared to NSTEMI/UA. Mortality during the first 6 months of follow-up was higher in octogenarians presenting with STEMI compared to NSTEMI/UA individuals; however, on longer follow-up, NSTEMI/UA patients tend to have higher mortality. This higher mortality in STEMI patients can be explained by the higher incidence of mechanical complications as well as cardiogenic shock in the acute stage of STEMI that are associated with high risk of mortality.^30,31^ In line with our results, the higher rate of MACCE in patients with STEMI after PCI compared to those with NSTEMI has been reported in previous studies.^32^ Bouisset et al compared short- and long-term mortality rates in STEMI and NSTEMI patients and showed that the short-term (28-day) mortality was significantly worse in STEMI patients; however, over longer follow-up periods NSTEMI patients had similar risk of death compared to STEMI patients.^33^ Another study conducted by Hirsch et al evaluated early and late outcomes in patients undergoing PCI and showed that patients with STEMI had a great risk of mortality during the first 6 months but survival was similar to those with NSTEMI/UA thereafter – up to 6 years.^34^

 Although invasive approaches such as PCI are associated with increased risk of adverse outcomes in the elderly, medical management in octogenarians was associated with 50% chance of later hospitalization and revascularization.^35^ Thus, it is reasonable to select patients and identify factors associated with PCI outcomes in octogenarians in order to achieve the optimal prognosis. Our findings emphasize the essential role of successfully performed PCI (defined as post-procedural TIMI flow 3) in old patients presenting with both STEMI or NSTEMI/UA. The prognostic impact of successful revascularization therapy in young patients has been proved in several randomized trials; but only few studies emphasized the importance of successful PCI in elderly patients with STEMI or NSTEMI/UA. Octogenarians have more complex lesions and higher prevalence of left main stenosis compared to the younger population and thus, undergo more complex PCI procedures.^12^ As we identified in our study, left main stenosis was associated with higher risk of MACCE in both STEMI and NSTEMI/UA patients.

 Similar to our findings, Miranda Malpica et al evaluated 73 patients ( ≥ 80 years) and showed that the predictors of 1-year MACE and mortality were pre- and post-operative TIMI flow < 3 and diabetes mellitus.^36^ However, in our study, only post-operative TIMI flow < 3 flow was associated with higher risk of MACCE. Bauer et al evaluated 47 407 patients undergoing PCI and identified the independent predictors of in-hospital mortality in those ≥ 75 years.^27^ Similar to our results, TIMI flow after PCI was associated with death after PCI. Another study conducted by Bromage et al analyzed 10 249 patients with STEMI treated with primary PCI and showed that 5-years mortality was significantly higher in patients ≥ 80 years.^37^ Moreover, the independent predictors of all-cause mortality in octogenarians were renal failure, ejection fraction < 30%, and TIMI flow 3. According to results from the Bremen STEMI Registry, successful PCI (defined as TIMI flow 2 or 3 after PCI) was associated with lower in-hospital and 1-year mortality in elderly patients presenting with STEMI; also, similar to our findings, diabetes mellitus was not a predictor of in-hospital mortality after PCI.^28^ Caretta et al evaluated 139 patients ( > 80 years) and showed that in univariate analysis, older age, TIMI flow < 3, diabetes, left main stenosis and left ventricular ejection fraction (LVEF) < 40% were associated with higher 1-year mortality; however, only TIMI flow < 3 and LVEF < 40% were the independent predictors of 1-year mortality.^38^ Batchelor et al evaluated 7472 octogenarians who underwent PCI and showed that diabetes mellitus, LVEF < 35%, renal failure, and cardiac shock were independent predictors of mortality.^7^ Moreover, in our study, dyslipidemia was associated with lower risk of MACCE in NSTEMI patients; one possible explanation for the protective effect of dyslipidemia might be that the use of lipid-lowering therapy such as statins before PCI is more likely in patients with dyslipidemia. The risk of all-cause mortality and MACE after revascularization has been shown to be lower in those receiving statin.^39,40^ Another explanation might be that patients with known dyslipidemia are considered high-risk and thus, they receive higher doses of statins and also, they are more expected to have regular exercise and healthy diets, to be aware of the risk of cardiovascular diseases, and to manage cardiovascular risk factors such as hypertension. However, this was beyond the scope of our study and further studies are needed to explain this possible protective effect.

###  Strength and Limitation 

 The present study has some possible limitations. Our results were based on short-term follow-up (median 12 months), and for more accurate results, further studies with longer follow-up periods are warranted. This a single-center study which should be taken into consideration when assessing the generalizability of our findings. However, THC is a referral educational university and admits patients from all over the country.

 The major strengths of this study are the high power of the study due to its large sample size and consequently, high prevalence of events. Moreover, we extracted our data from the THC registry databank, which records the individuals’ data prospectively.

## Conclusion

 In this registry-based study, we found that MACCE and mortality were higher in octogenarians who underwent PCI, compared to younger adults. Moreover, in our octogenarian patient cohort, the risk of MACCE was higher in those presenting with STEMI compared to NSTEMI/UA and the mortality trend was similar in the first 6 months; however, the 1-year trend was in favor of the STEMI subgroup. Our data also provided insights into the independent predictors of MACCE in octogenarians after 1-year follow-up.

## Supplementary Materials

Supplementary file 1 contains Tables S1-S2 and Figure S1.
