# Supplementary material for: Outcome of Percutaneous Coronary Intervention in Old Patients Presenting with Acute Coronary Syndrome
Source: Arch Iran Med. 2022 Aug 1;25(8):523–32. doi: 10.34172/aim.2022.84 (PMC13661076; doi:10.34172/aim.2022.84)
Supplement: Supplementary file 1 [file aim-25-523-s001.pdf]

Arch Iran Med. August 2022;25(8):523-532
10.34172/aim.2022.84

Outcome of Percutaneous Coronary Intervention in Old Patients Presenting with Acute Coronary Syndrome

Aida Fallahzadeh, MD[1,2]; Ali Sheikhy, MD[1,2]; Afsaneh Aein, MD[3]; Mojtaba Salarifar, MD[4]; Hamidreza Pourhosseini, MD[4]; Hassan Aghajani, MD[4]; Mohammad Alidoosti, MD[4]; Saeed Sadeghian, MD[4]; Kaveh Hosseini, MD[4*]

[1]Tehran Heart Center, Tehran University of Medical Sciences, Tehran, Iran
[2]Non-Communicable Disease Research Center, Endocrinology and Metabolism Population Sciences Institute, Tehran University of Medical Sciences, Tehran, Iran
[3]Department of Health Education and Health Promotion, School of Public Health, Iran University of Medical Sciences, Tehran, Iran
[4]Department of Cardiology, Tehran Heart Center, Tehran University of Medical Sciences, Tehran, Iran

Supplementary file 1

Table S1. Estimated Hazard ratios (HRs) of MACCE predictors by univariate analysis in octogenarians

**Predictors**	**STEMI**		**NSTEMI/UA**	
	**HR (95% CI)**	***P***	**HR (95% CI)**	***P***
Age	1.04 (0.99–1.09)	0.107	1.02 (0.94–1.12)	0.560
BMI (Kg/m^2)	1.034 (0.98–1.08)	0.185	0.97 (0.91–1.04)	0.458
Family history	1.08 (0.47–2.48)	0.863	0.74 (0.26–2.08)	0.569
Dyslipidemia	0.99 (0.62–1.56)	0.955	0.45 (0.24–0.85)	0.014
Female	0.967 (0.61–1.54)	0.884	0.84 (0.44–1.60)	0.590
Hypertension	0.98 (0.62–1.55)	0.919	1.01 (0.56–1.88)	0.965
Diabetes mellitus	1.63 (1.04–2.56)	0.033	1.39 (0.77–2.51)	0.280
Smoking	0.94 (0.67–1.34)	0.751	1.16 (0.78–1.72)	0.478
Opium consumption	0.76 (0.30–1.96)	0.671	0.36 (0.06–2.38)	0.292
Cerebrovascular disease	1.04 (0.42–2.56)	0.941	0.54 (0.13–2.28)	0.405
COPD	0.31 (0.04–2.21)	0.241	2.60 (0.62–10.89)	0.191
LVEF	0.98 (0.96–1.00)	0.285	0.92 (0.97–1.01)	0.223
eGFR (mL/min)	0.98 (0.96–0.99)	0.034	0.97 (0.95–0.99)	0.007
Multivessel disease	1.05 (0.64–1.74)	0.840	1.80 (0.81–4.01)	0.150
Left main stenosis	1.85 (1.04–3.31)	0.037	2.27 (1.11–4.67)	0.025
Stent length (mm)	0.99 (0.97–1.03)	0.948	1.00 (0.96–1.04)	0.943
Preprocedural TIMI flow 3	0.58 (0.28–1.22)	0.153	0.65 (0.28–1.48)	0.303
Postprocedural TIMI flow 3	0.35 (0.16–0.76)	0.008	0.29 (0.11–0.80)	0.016
ACC/AHA type C	1.14 (0.78–1.69)	0.493	1.16 (0.63–2.14)	0.636

Yellow highlight: Candidate to enter the multivariable analysis
Diabetes in STEMI: HR 1.45 (95%CI: 0.89–2.34), *P* = 0.128: All mentioned predictors in the multivariate analysis were hypothetically associated with MACCE; hence, diabetes in ST-elevated patients did not have a significant p-value (*P* = 0.128), and did not cause more than 10% change in the final model. Accordingly, it was excluded from the final predictors.

Table S2. Estimated Hazard ratios (HRs) of MACCE predictors by multivariate analysis in octogenarians

**Predictors**	**STEMI****		**NSTEMI/UA*****	
	**HR (95% CI)**	***P***	**HR (95% CI)**	***P***
eGFR	0.98(0.96–0.99)	0.029	0.97 (0.95–0.99)	0.006
Left main stenosis*	1.84 (0.92–3.66)	0.081	2.50 (1.17–5.34)	0.018
Postprocedural TIMI flow 3	0.29 (0.16–0.53)	<0.001	0.22 (0.10–0.49)	<0.001
Dyslipidemia	—	—	0.37 (0.19–0.72)	0.003

*Left main stenosis: mentioned variable, did not have significant p-value in the multivariate analysis, yet it caused more than 10% alteration in the final model, thus, "Left main stenosis" considered as the independent predictor.
**PH assumption: *P*-value = 0.550
***PH assumption: *P*-value = 0.982

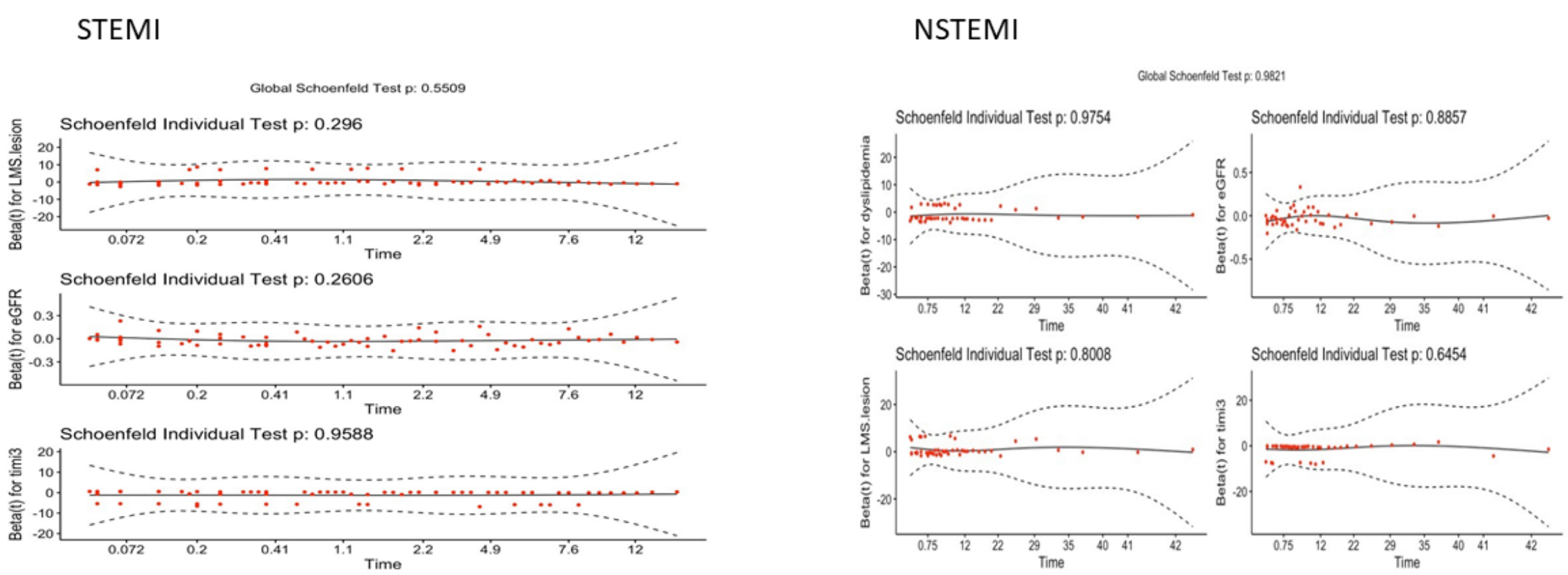

Figure S1. Assessment of proportional hazard assumption for Figure 4.
